# The link between diabetes and atrial fibrillation: cause or correlation?

**DOI:** 10.4103/0975-3583.59978

**Published:** 2010

**Authors:** Yihong Sun, Dayi Hu

**Affiliations:** *Heart Centre, Peking University People’s Hospital, China yihongsun72@163.com*

Atrial fibrillation (AF) is the most common form of arrhythmia in the world. As the population ages, it is estimated that the prevalence of AF will increase by 2.5 fold in the next 50 years.^1^ At the same time, diabetes has become a pandemic disease in the western world as well as in developing countries. Independent risk factors for chronic AF include hypertension, heart failure, valvular heart disease and cardiomyopathy. The development of AF is likely to be multifactorial and the mechanism is elusive, while there is emerging evidence on the correlation between AF and diabetes mellitus (DM). DM and AF share common antecedents such as hypertension, atherosclerosis and obesity. Population-based studies suggested that DM is an independent risk factor for atrial fibrillation.^2^ Both DM and AF are marked predictor for stroke and mortality. The causal relation between DM and AF is still debatable and will be discussed.

## DM as risk factor for AF

DM is one of the most common concomitant diseases in patients with AF.^3^ In a survey of hospitalized patient, AF occurred in 14.9% of DM patients vs 10.3% in control group with hypertension but no DM.^4^ DM has been cited as the risk factor for AF for decades. In the early 1990s, the Framingham study indicated DM to be an independent risk factor for AF with OR of 1.4 for men and 1.6 for women after 38 years followup.^2^ In the analysis of 41436 residents in Japan, the prevalence of DM in AF patients is higher than in controls (20% vs 12%). The multivariate analysis showed that DM is independently associated with AF (OR, 1.46).^5^ In the cross sectional survey in mainland China, the prevalence of AF in participants with self reported DM is higher than those without known DM (1.29% vs 0.88%) after adjustment for age and sex.^6^ However, in the development of risk score for AF, DM was not a significant predictor of AF in the Framingham cohort.^7^ Data of China survey showed that DM is not an independent risk factors for AF in the multivariate model.^6^

In the observational cohort study of DM registry, the incidence of AF in diabetic patients was much higher than in controls (9.1% vs 6.6%) over a mean follow-up period of of 7.2 years. After adjustment for other risk factors, DM was associated with a 26% increased risk of AF among women (HR, 1.26), but not a statistically significant factor among men.^5^ In patients with hypertension, DM is not an independent predictor for new onset AF based on the post hoc analysis from ALLHAT (Antihypertensive and lipid-Lowering treatment to prevent heart attack trial).^9^ However, an retrospective analysis of VALUE (Valsartan antihypertensive long-term use evaluation) study showed hypertensive patients with new onset DM had a significantly higher event rate of new onset AF compared with those without DM (5.4% vs 3.8%, HR=1.49) even adjusted for body mass index.^10^

## Mechanism of the DM related AF

The link between DM and AF in epidemiology needs to be confirmed in large, prospective, long term study, although there were evidences showing the underlying biological interlink. Studies have indicated that inflammation might play a role in the generation, maintenance, and perpetuation of AF, although the mechanism of AF is still elusive. In atrial biopsies of patients with lone AF, there were increased CRP and IL-6and marked inflammatory infiltrates. 11–12 CRP was two-fold higher in AF patients compared with control group without atrial arrhythmia. It also has been demonstrated that inflammatory response may underlie the pathologic processes of DM.

Glucose and insulin disturbance can directly affect the myocardium in atrium and ventricle, leading to AF. Left ventricular (LV) hypertrophy has been associated with DM and abnormal glucose tolerance in several epidemiology studies. LV hypertrophy is a significant risk factor for AF. Analysis of the Framingham study subjects showed that LV mass increased with the worsening of glucose tolerance and the trend was more striking in women than in man. There were also close relationship between insulin resistance and LV mass, as well as LV wall thickness, in women both with normal and abnormal glucose tolerance.^13^ Prospective data from large population based studies has established the relationship between LA size and risk of developing AF. In the same study, LV size also increased with glucose tolerance and insulin resistance in both sexes, but this relation was largely accounted for by obesity. The association between AF and DM in relation to body size needs further investigation.

Several observations suggest that the automatic nerve system plays an important role in both the initiation and/ or the maintenance of AF in humans. In most patients with organic heart diseases, the paroxysmal AF episodes appear more sympathetically dependent. In the animal model of DM, the occurrence of AF was enhanced by adrenergic activation in diabetic heart, in which the sympathetic innervations was evident.^14^–^15^ The intra –atrial conduction delay and fibrotic deposition in atria play a major role in producing atrial tachyarrhythmia in diabetes animal model. The heterogeneous increase in sympathetic innervations was proved to be associated with the promotion of AF in several studies.

## Summary

In conclusion, the association between AF and DM has been proved both in epidemiology and experimental studies. The causal links need to be verified in large cohort study. A recent report from NIH stated that AF prevention has received less attention than treatment and there were great knowledge gaps in the understanding of risk factors of AF.^16^ One important research area may be the impact of DM on the incidence, recurrence and progression of AF.
